# Alveolar instability (atelectrauma) is not identified by arterial oxygenation predisposing the development of an occult ventilator-induced lung injury

**DOI:** 10.1186/s40635-015-0054-1

**Published:** 2015-06-09

**Authors:** Penny L Andrews, Benjamin Sadowitz, Michaela Kollisch-Singule, Joshua Satalin, Shreyas Roy, Kathy Snyder, Louis A Gatto, Gary F Nieman, Nader M Habashi

**Affiliations:** Department of Surgery, SUNY Upstate Medical University, 750 East Adams Street, Syracuse, NY 13210 USA; Department of Critical Care, R Adams Cowley Shock Trauma Center, Baltimore, MD USA; Department of Biology, SUNY Cortland, Cortland, NY USA

## Abstract

**Background:**

Improperly set mechanical ventilation (MV) with normal lungs can advance lung injury and increase the incidence of acute respiratory distress syndrome (ARDS). A key mechanism of ventilator-induced lung injury (VILI) is an alteration in alveolar mechanics including alveolar instability or recruitment/derecruitment (R/D). We hypothesize that R/D cannot be identified by PaO_2_ (masking *occult VILI*), and if protective ventilation is not applied, ARDS incidence will increase.

**Methods:**

Sprague-Dawley rats (*n* = 8) were anesthetized, surgically instrumented, and placed on MV. A thoracotomy was performed and an in vivo microscope attached to the pleural surface of the lung with baseline dynamic changes in alveolar size during MV recorded. Alveolar instability was induced by intra-tracheal instillation of Tween and alveolar R/D identified as a marked change in alveolar size from inspiration to expiration with increases in positive end-expiratory pressure (PEEP) levels.

**Results:**

Despite maintaining a clinically acceptable PaO_2_ (55–80 mmHg), the alveoli remained unstable with significant R/D at low PEEP levels. Although PaO_2_ consistently increased with an increase in PEEP, R/D did not plateau until PEEP was >9 cmH_2_O.

**Conclusions:**

PaO_2_ remained clinically acceptable while alveolar instability persisted at all levels of PEEP (especially PEEP <9 cmH_2_O). Therefore, PaO_2_ levels cannot be used reliably to guide protective MV strategies or infer that VILI is not occurring. Using PaO_2_ to set a PEEP level necessary to stabilize the alveoli could underestimate the potential for VILI. These findings highlight the need for more accurate marker(s) of alveolar stability to guide protective MV necessary to prevent VILI.

## Background

The acute respiratory distress syndrome (ARDS) remains a serious clinical challenge affecting nearly 200,000 patients annually, claiming the lives of 30–40 % of those afflicted [1]. Although mechanical ventilation (MV) is applied to patients without lung injury, improperly set MV [2] can cause a secondary ventilator-induced lung injury (VILI) [3], playing a key role in the high incidence of ARDS (25 %) within 48 h of MV initiation [4, 5]. Most cases of ARDS evolve over 30–72 h after hospital admission where lung protective strategies are typically implemented *after* the development of lung injury and clinically diagnosed by a deterioration of oxygenation characterized by a decline in PaO_2_/FiO_2_ (P/F) ratio [6, 7]. Although there are alternative markers of optimizing mechanical ventilation, most definitions of ARDS [6, 7] rely on oxygenation (PaO_2_) as a marker of pulmonary function. Lung protection is generally instituted in a *reactive* rather than *proactive* approach with an emphasis on *treating* rather than *preventing* the progression of worsening lung injury. Further, clinicians target PaO_2_ or saturation (SpO_2_) [8] to determine if ventilator strategies are effective prior to and after the development of acute lung injury (ALI). However, reliance on PaO_2_ as a marker of lung function presumes there is no lung injury prior to ARDS perpetuating a binary concept of ARDS. A key to understanding lung-ventilator interactions may lie in sub-clinical mechanisms of lung injury and the impact of MV on the “micro-environment” at the alveolar/alveolar duct level rather than centering on the “macro-environment” and parameters displayed on the ventilator (i.e., tidal volume (Vt), plateau pressure) using PaO_2_ and SpO_2_.

Recently, it has been suggested that the primary mechanism of VILI is tidal alveolar recruitment/derecruitment (R/D) [9, 10]. Our group has shown that tidal R/D leads to persistent alveolar instability causing severe histologic lung injury and ARDS [11] and optimizing alveolar stability is critical to preventing VILI and limiting ARDS [12–14]. PaO_2_ may not be an accurate surrogate of alveolar stability; therefore, preventing ARDS using preemptive MV requires clinicians to consider the potential for *occult alveolar instability* (i.e., R/D without a significant fall in PaO_2_) [6]. In this study, we hypothesized that in an acutely injured lung, alveolar instability would persist despite improvements in PaO_2_ with increasing levels of positive end-expiratory pressure (PEEP). Lack of correlation between PaO_2_ and alveolar stability would provide a mechanistic explanation of why patients with normal PaO_2_ evolve to ARDS after 48 h of MV [4, 15]. In this study, we show direct visual evidence using in vivo microscopy that alveolar R/D cannot be predicted using PaO_2_ as a surrogate for alveolar stability and protective MV.

## Methods

### Vertebrate animals

The Institutional Animal Care and Use Committee at Upstate Medical University, Syracuse, NY, approved the studies. All experiments were performed in adherence to the guidelines established by the National Institutes of Health for the use of experimental animals in research.

### Surgical preparation

Adult male Sprague-Dawley rats (*n* = 8) weighing 590 ± 19.2 g were anesthetized via an intraperitoneal injection of ketamine/xylazine (90 and 10 mg/kg, respectively) dosed at 0.1 mg/kg of ketamine before surgery as well as during the experiment to provide adequate anesthesia. To ensure that the Vt remained constant, the animals were paralyzed with rocuronium to inhibit spontaneous breathing. A tracheostomy was performed, and time-cycled/pressure-controlled mechanical ventilation was initiated with a PEEP of 3 cmH_2_O, pressure control (*P*_control_) of 15 cmH_2_O, respiratory rate of 30 breaths/min, and FiO_2_ of 100 % (Hamilton G5 ventilator, Hamilton Medical Inc., Reno, NV). A jugular vein was cannulated for the administration of fluids and a carotid artery catheter placed for blood gas samples and hemodynamic monitoring.

### Hemodynamic, pulmonary, and blood gas measurements

Hemodynamic and pulmonary parameters were continuously monitored (Philips Healthcare and Hamilton G5 ventilator). Arterial blood gas was sampled at baseline and each PEEP level, as defined in “Lung injury,” to record pH, PaCO_2_, PaO_2_, and PaO_2_/FiO_2_ (P/F) ratios (cobas b221, Roche Diagnostics).

### Lung injury

Serial blood gases were taken and the respiratory rate adjusted until PaCO_2_ was between 35 and 45 mmHg. A baseline blood gas was performed and lung injury induced via 0.2 % Tween-20 in normal saline (16 cc/kg) instillation via an endotracheal tube. This was repeated until a P/F <100 mmHg was reached.

### In vivo microscopy

After induction of lung injury, a right thoracotomy was performed to expose the pleural surface of the right lung. The in vivo microscope (epi-objective microscope with epi-illumination; Olympus America Inc.) was approximated to the nondependent (anterior) portion of the right lung and 5 cmH_2_O suction applied to capture a steady field of the alveoli at ×10 magnification. Although dependent lung regions may be more susceptible to R/D, stability in dependent regions would be unlikely if alveolar stability in nondependent regions was not achieved. In this model, we used individual rats as their own control and documented alveolar stability at baseline. After Tween injury, alveolar behavior became unstable demonstrating R/D. Thirty seconds of video was recorded from an attached digital video camera (Sony CCD color video camera SSC-S20) and uploaded to a computer while maintaining a PEEP of 3 cmH_2_O and *P*_control_ of 15 cmH_2_O. Subsequently, PEEP was increased in 3 cmH_2_O increments and videos recorded from the in vivo microscopy at each setting until a PEEP of 18 cmH_2_O was achieved. This procedure was repeated at each PEEP level in five distinct parenchymal areas of the right nondependent lung.

### Image capture and processing

Video was captured using Pinnacle Studio software (Corel Corp.) for analysis of alveolar mechanics. Individual frames were extracted and analyzed at the plateau pressure (*P*_plateau_) and peak expiration (PEEP) for five lung fields per PEEP level. The alveoli were identified in the frames and manually outlined using Photoshop CS6 (Adobe Inc.) with areas calculated using Image-Pro software (Media Cybernetics). The areas calculated represent alveolar inflation at end-inspiration (I) and end-expiration (E) and are each represented as a fraction of the total microscopic lung field. Alveolar instability was assessed by comparing the percent difference between I and E (%ΔI-E). A greater %ΔI-E is suggestive of increased R/D and alveolar instability.

### Statistics

Least squares regression was used to determine whether alveolar stability and arterial oxygenation were linearly related (Microsoft Excel, 2008).

## Results

### Physiologic parameters

All animals displayed severe lung injury after Tween instillation with average P/F ratio <100 mmHg. As PEEP was increased, there was a concomitant decrease in mean arterial pressure but no change in heart rate (Table 1). With increasing PEEP, there was no significant change in pH but there was a decrease in pCO_2_ with PEEP above 6 cmH_2_O. Increasing PEEP led to a correspondent increase in the mean airway pressure (Paw) and *P*_plateau_. Tidal volumes were set at 6 cc/kg, and minute ventilation was similar between groups (Table 2).

**Table 1:** Hemodynamic and blood gas measurements

	BL-uninjured	Post-injury	PEEP 3	PEEP 6	PEEP 9	PEEP 12	PEEP 15	PEEP 18
HR (beats/min)	189.1 ± 59.1	186.4 ± 45.1	204.9 ± 44.5	172.2 ± 51.1	186.0 ± 38.3	176.7 ± 44.6	179.9 ± 61.5	150.5 ± 42.9
MAP (mmHg)	71.8 ± 21.1	61.3 ± 12.6	50.6 ± 16.2	52.5 ± 26.5	49.0 ± 24.1	47.4 ± 18.1*	44.5 ± 16.5*	39.1 ± 15.7*
pH	7.35 ± 0.07	7.246 ± 0.087	7.204 ± 0.049*	7.153 ± 0.101*	7.209 ± 0.066*	7.197 ± 0.109*	7.21 ± 0.15*	7.199 ± 0.195*
PaCO_2_ (mmHg)	38.11 ± 11.67	53.58 ± 14.43*	47.42 ± 13.21	49.68 ± 11.22	36.49 ± 4.85	35.43 ± 5.99	32.0 ± 7.0	29.39 ± 7.17
PaO_2_ (mmHg)	467.5 ± 163.5	54.03 ± 24.70*	60.07 ± 13.10*	106.3 ± 90.47*	169.7 ± 143.5*	221.5 ± 115.5*	266.0 ± 122.0*	352.0 ± 159.0
FiO_2_ (%)	100	100	100	100	100	100	100	100
% Saturation	97.63 ± 1.26	56.43 ± 20.96*	64.76 ± 16.04*	65.12 ± 17.54	88.77 ± 5.74	92.26 ± 8.58	93.15 ± 8.11	95.1 ± 5.98
P/F	507.6	54.03*	60.07*	106.3*	169.7*	221.5*	266.0*	352.0*

*p ≤ 0.05 as compared to baseline (BL)

**Table 2:** Pulmonary measurements

	BL-uninjured	Post-injury	PEEP 3	PEEP 6	PEEP 9	PEEP 12	PEEP 15	PEEP 18
Vt (ml)/kg	6.0 ± 0.5	5.9 ± 0.3	6.0 ± 0.5	5.9 ± 0.3	5.9 ± 0.3	5.9 ± 0.3	5.9 ± 0.3	5.875 ± 0.354
*P*_control_ (cmH_2_O)	12.6 ± 2.5	16.9 ± 1.2*	17.6 ± 2.2*	16.6 ± 2.5*	15.5 ± 3.1	14.9 ± 3.1	14.7 ± 3.9	12.88 ± 5.0
*P*_peak_ (cmH_2_O)	15.7 ± 2.6	20.1 ± 1.4*	20.7 ± 2.3*	22.5 ± 2.3*	24.5 ± 3.1*	27.0 ± 3.2*	29.7 ± 3.9*	30.88 ± 5.0*
*P*_plateau_ (cmH_2_O)	15.7 ± 2.6	20.0 ± 1.3*	20.6 ± 2.2*	22.6 ± 2.5*	24.5 ± 3.1*	27.0 ± 3.2*	29.7 ± 3.9*	30.88 ± 5.0*
*P*_mean_ (cmH_2_O)	6.59 ± 0.65	7.72 ± 0.43	7.94 ± 0.72	10.15 ± 0.95*	11.99 ± 1.35*	13.8 ± 1.9*	15.3 ± 3.6*	14.63 ± 2.8*
MV (l/min)	0.183 ± 0.009	0.187 ± 0.022	0.185 ± 0.022	0.184 ± 0.026	0.188 ± 0.019	0.187 ± 0.016	0.19 ± 0.028	0.175 ± 0.018
RR (breaths/min)	30.5 ± 1.6	30.4 ± 2.0	30.9 ± 2.5	32.0 ± 3.3	32.0 ± 3.3	31.6 ± 2.5	31.4 ± 2.9	30.13 ± 3.23

*p ≤ 0.05 as compared to baseline (BL)

### Alveolar stability and PaO_2_

**Fig. 1:**
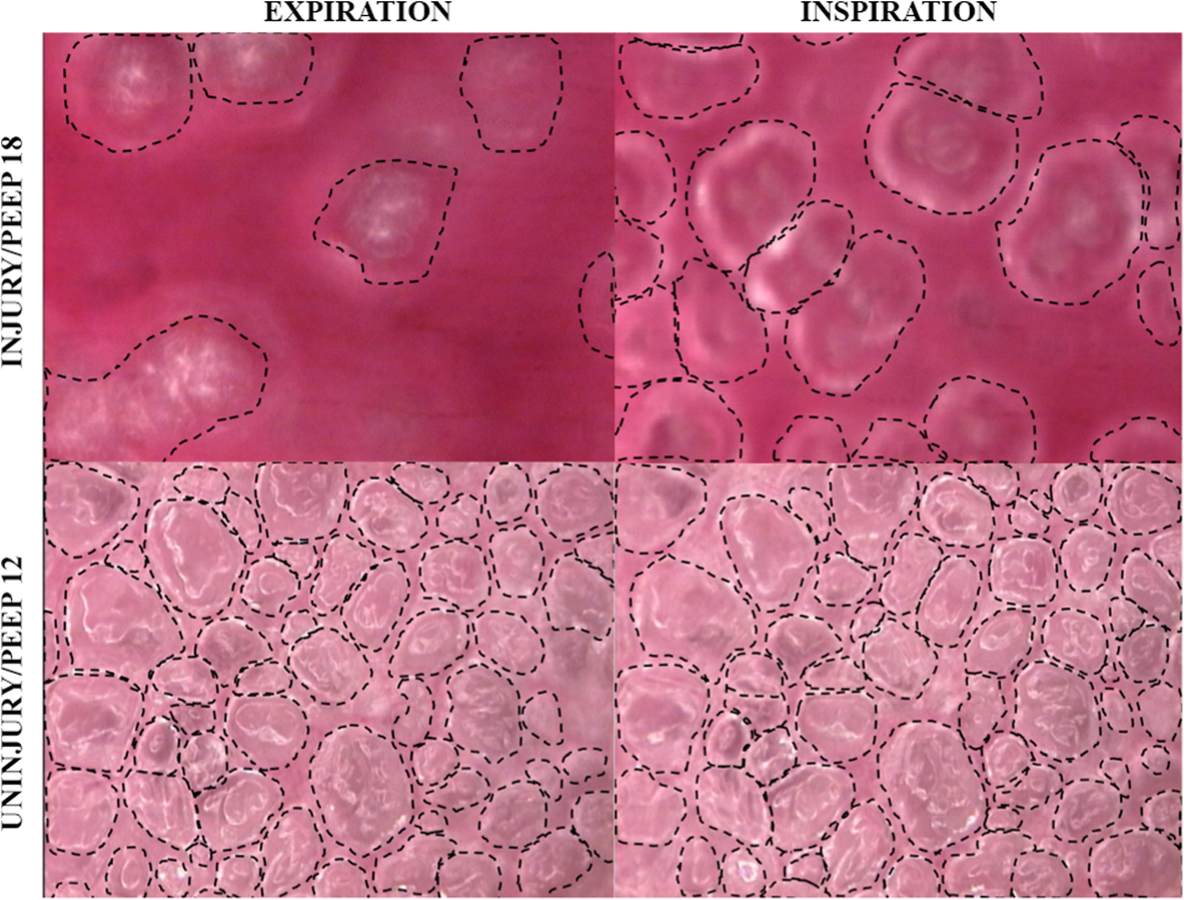
In vivo microscopy showing alveolar inflation of non-injured (PEEP 12) and injured (PEEP 18) lungs at end-inspiration and end-expiration

Subpleural alveoli at I and E in both the uninjured and injured lungs are seen in Fig. 1 with individual alveoli outlined in *dots*. Measuring alveolar size change between I and E is used to assess alveolar stability.

### Uninjured alveolar stability and PaO_2_

There was a minimal size change during tidal ventilation in the normal lung with 12 cmH_2_O of PEEP compared to a large change in alveolar size in the injured lung indicating severe alveolar instability even with 18 cmH_2_O of PEEP (Fig. 1).

### Injured alveolar stability and PaO_2_

**Fig. 2:**
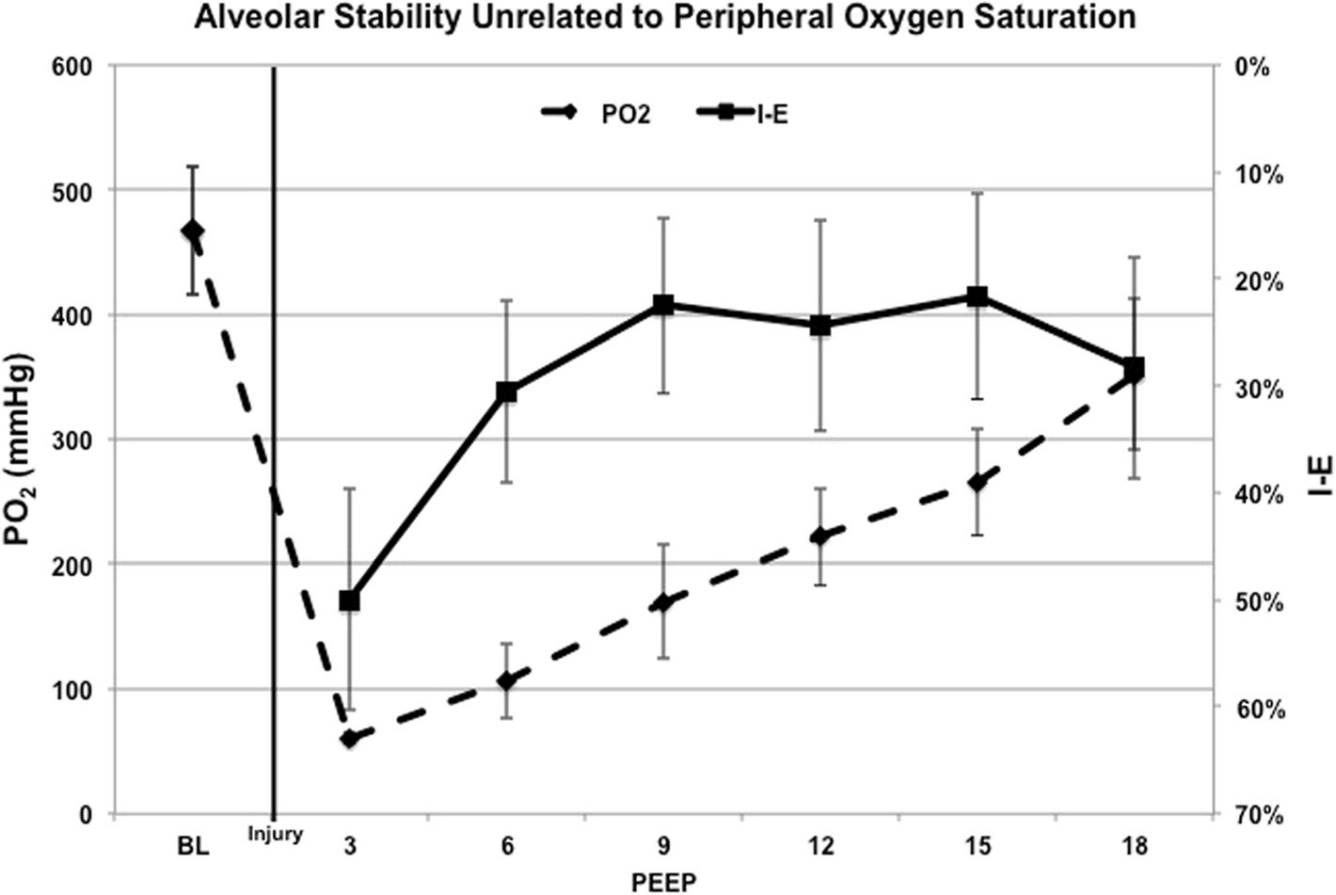
Stepwise increases in low levels of PEEP (3–9 cmH_2_O) led to an increase in both alveolar stability and arterial oxygenation as measured by pO_2_. High levels of PEEP (12–18 cmH_2_O), however, improved arterial oxygenation in a stepwise fashion without a concomitant increase in alveolar stability

Examination of the aggregate data shows that a stepwise increase in PEEP (3–9 cmH_2_O) led to an increase in both alveolar stability and PaO_2_ (Fig. 2). However, further increased levels of PEEP (12–18 cmH_2_O) improved PaO_2_ without a concomitant increase in alveolar stability (Fig. 2). Examination of individual animals demonstrated that PaO_2_ remained largely unchanged despite a spectrum of alveolar instability at a given PEEP level.

## Discussion

**Fig. 3:**
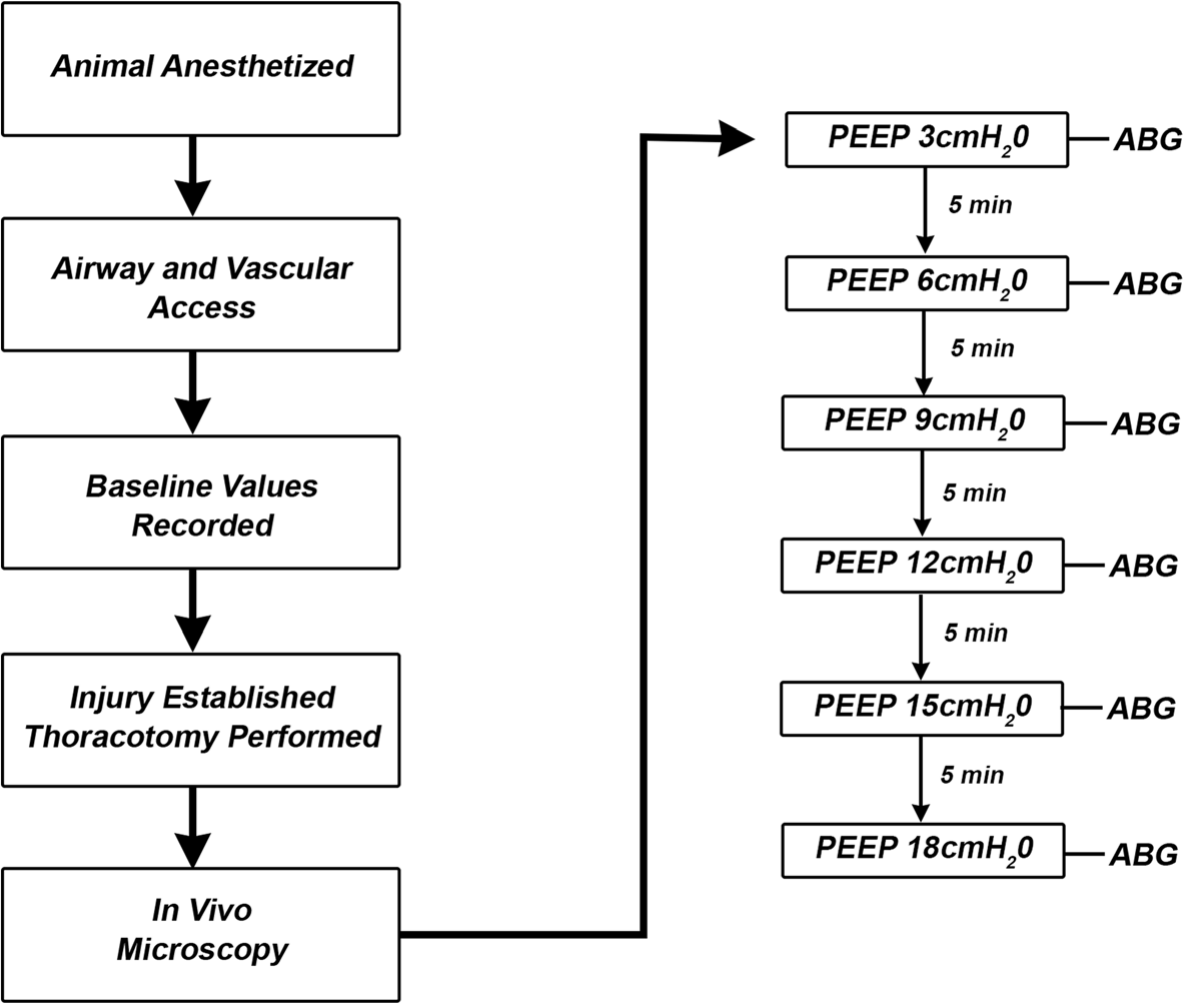
Experimental timeline: Following instrumentation, lung injury, and thoracotomy, PEEP titrations were \incrementally increased by 3 cmH_2_O until 18 cmH_2_O was reached. Arterial blood gas (ABG) and in vivo microscopy at inspiration and expiration was recorded after a 5-min equilibration

The most important findings of this study were as follows: (a) unstable alveoli oxygenated blood to nearly the same PaO_2_ as stable alveoli, particularly at levels of PEEP of 3–9 cmH_2_O; (b) alveolar stability did not improve when PEEP was increased above 9 cmH_2_O despite continual improvement in PaO_2_ up to PEEP of 18 cmH_2_O (Fig. 3); and (c) combined with our prior work, this shows that PEEP may have a greater effect recruiting and distending conducting airways rather than the alveoli [16]. In a previous study, we showed increasing levels of PEEP increases micro-strain of the conducting airways with less effect on alveolar area [16]. In the present study, low levels of PEEP may have recruited collapsed conducting airways initially stabilizing communicating alveoli (steep portion of alveolar stability curve in Fig. 2). However, an increase in PEEP may have caused progressive distention in the conducting airways rather than increasing alveolar area and stability. Despite increasing PEEP, alveolar stability plateaus above PEEP of 9 cmH_2_O and never reaches the complete stability (i.e., R/D = 0 %) of the uninjured lung [17]. These data suggest that alveolar stability has a nonlinear relationship to PaO_2_.

We hypothesize that improvement in PaO_2_ was due to end-inspiratory tidal recruitment of collapsed alveoli rather than recruitment of unstable alveoli throughout the ventilator cycle (i.e., both I and E). Oxygen would be exchanged during inspiration as the alveolus “pops” open and coupled with the rapid transit time of the erythrocyte, maintaining a relatively normal PaO_2_; however, the alveoli collapse during expiration. The linear improvement in PaO_2_ without a corresponding increase in alveolar stability suggests persistent alveolar instability, and the potential to expose the lung to *occult VILI* exists and may silently progress to clinical ARDS.

Our data and others demonstrate that perhaps the most widely used marker for lung injury [PaO_2_] is insensitive to detect alveolar instability. Baumgardner et al. used a high-speed intravascular PaO_2_ sensor in a saline lavage ARDS model and found that PaO_2_ fluctuated within the respiratory cycle [18]. They postulated that PaO_2_ rose during inflation when the alveoli were open and fell (during exhalation) when the alveoli collapsed [18]. Using a fiber optic oxygen sensor that detects rapid PaO_2_ changes, Formenti and colleagues documented PaO_2_ fluctuations between 37 and 375 mmHg between the I and E cycles as a result of cyclical atelectasis [19]. Caltabeloti et al. have shown, using ultrasound, that lung aeration decreases during fluid loading without any deterioration in PaO_2_. These data demonstrate that although oxygenation improves, alveolar R/D-induced VILI may persist but cannot be identified by standard clinical measurements (i.e., PaO_2_). Therefore, if solely guided by PaO_2_, ventilator adjustments may aggravate alveolar instability, particularly when lung injury is evolving secondary to volume resuscitation and a progressing systemic inflammatory response syndrome (SIRS) [20]. Furthermore, uncontrolled R/D increases lung inflammatory response and may propagate other organ dysfunctions and multisystem organ failure [21]. This potentially explains the dichotomy of results in the ARDSnet trial (i.e., improved oxygenation in the high Vt/end-inspiratory pressure group, yet an increase in mortality) [22]. The low tidal volume strategy with lower end-inspiratory pressure may have resulted in a greater regional lung collapse and lower oxygenation; but because these regions remain collapsed throughout the breathing cycle, they are protected from tidal R/D, resulting in less alveolar instability rather than overdistension (Fig. 2) [23].

Although recruitment maneuvers (RM) were not used in this study, previous work from this lab using in vivo microscopy demonstrated that the alveoli open following an RM but remain unstable unless combined with sufficient amounts of PEEP to maintain alveolar patency [24]. Oxygenation significantly improves following RMs despite persistent alveolar R/D, suggesting that unstable alveoli participate in oxygenation during the portion of the ventilatory cycle in which they are open [11]. RMs improve oxygenation but have not demonstrated a mortality benefit [25, 26] supporting our postulate that despite improved PaO_2_, alveolar instability may persist causing progressive lung injury. Nontraditional approaches to MV such as high-frequency oscillatory ventilation (HFOV) use similar goals of PaO_2_ or SpO_2_ to guide adjustment of ventilator settings. Two recent trials used oxygenation triggers to adjust Paw and the decision to convert from HFOV back to conventional ventilation [27, 28]. However, experimentally, we have shown that adjusting Paw during HFOV based on PaO_2_-guided protocols resulted in significantly greater histologic lung injury as compared with maintaining a constant Paw after lung injury [27, 28]. Combined, these data support our hypothesis that PaO_2_ does not correlate with alveolar stability with enough sensitivity to guide ventilator adjustments and relying solely on oxygenation to make ventilator adjustments could increase alveolar instability and promote occult VILI.

Clinically, these data may be important as discordance between PaO_2_ and the degree of alveolar instability creates the potential for occult R/D-induced VILI. Robust clinical markers for evaluating alveolar stability are lacking; thus, VILI remains a genuine concern among clinicians who intend to minimize iatrogenic lung injury during MV. In clinical practice, PaO_2_ or SpO_2_ is routinely used to guide ventilator adjustments [8]. The commonly used PEEP/FiO_2_ table derived from the ARDSnet strategy links a PaO_2_ or SpO_2_ goal to regulate PEEP and FiO_2_. Several investigators have suggested that the PEEP/FiO_2_ scale does not impose a physiologic end-point for clinical or computed tomography-guided evidence of recruitment and alveolar stability [29, 30]. In addition, clinicians frequently underdiagnose ARDS suggesting that acceptable oxygenation may leave many without concern for ongoing lung injury [31]. In this study, all animals had an acceptable P/F ratio based on the PEEP/FiO_2_ table despite a 25–60 % increase in alveolar instability from baseline. This suggests that achieving clinically acceptable oxygenation goals may not equate with decreased alveolar instability, allowing persistent R/D and the potential for VILI.

### Summary

This study has identified the need of detecting alveolar instability and optimizing MV settings to promote alveolar stability and lung homogeneity using methods other than PaO_2_. Possible methods include the following: (a) electrical impedance tomography (EIT) which provides an assessment of lung volume, PEEP level, and Vt distribution rather than the relationship of Vt to ideal body weight [32–34]; (b) routine evaluation of the respiratory system as a whole with transpulmonary pressure measurement to determine the effect of the chest wall and abdomen on lung mechanics [35–37]; and (c) monitoring the expiratory flow graphics when using modes such as airway pressure release ventilation (APRV) to control expiratory time constants which has been shown to eliminate alveolar instability [38, 39]. These methods are all currently available and may guide ventilator adjustments rather than relying on the insensitivity of PaO_2_ and SpO_2_ or the macro-environment parameter such as Paw [14, 32–39].

The concept that preemptive ventilation strategies may be therapeutic is obverse to the current understanding that MV creates or aggravates lung injury (i.e., VILI). Although both concepts may be accurate, the key may be the method of MV and the timing of application in the pathogenesis of the disease (i.e., early vs. late) [13, 14, 39, 40]. The causal role of MV in progressing acute lung injury (ALI) may be determined by adjustment of ventilator settings to adequately prevent alveolar instability and heterogeneity. If adjusted properly, the ventilator may be used to halt the progression of ALI pathogenesis and reduce the incidence of ARDS [12–14, 16, 39, 40].

### Critique of the model

While we speculate that the steady increase observed in PaO_2_ without a marked change in alveolar stability at PEEP levels of >9 cmH_2_O was likely due to increased alveolar recruitment, this recruitment was not confirmed by direct measurement. Also, our in vivo microscope has limitations such as the shallow depth of field (70 μm), limiting observations of alveolar mechanics to only two dimensions. We used gentle suction (≤5 cmH_2_O) to hold lung tissue in place underneath the glass coverslip in all experimental settings standardizing this artifact, which has been shown previously to have minimal effect on alveolar mechanics [41].

## Conclusions

Our data suggest that oxygenation (PaO_2_) may be a poor marker for alveolar stability and occult VILI may exist despite meeting acceptable clinical criteria. Further, using PaO_2_ to guide titration of MV settings (PEEP and Pplat) may result in increased alveolar instability and increase the potential for VILI. New methods to identify alveolar stability are needed for clinical assessment and protection from occult VILI. In combination with judicious use of MV, the goal of eliminating VILI in the critical care population may eventually be realized.

## References

[1] Goss CH, Brower RG, Hudson LD, Rubenfeld GD, Network A (2003). Incidence of acute lung injury in the United States. Crit Care Med.

[2] Whitehead T, Slutsky AS (2002). The pulmonary physician in critical care * 7: ventilator induced lung injury. Thorax.

[3] Slutsky AS, Ranieri VM (2013). Ventilator-induced lung injury. N Engl J Med.

[4] Gajic O, Dara SI, Mendez JL, Adesanya AO, Festic E, Caples SM, Rana R, St Sauver JL, Lymp JF, Afessa B, Hubmayr RD (2004). Ventilator-associated lung injury in patients without acute lung injury at the onset of mechanical ventilation. Crit Care Med.

[5] Shari G, Kojicic M, Li G, Cartin-Ceba R, Alvarez CT, Kashyap R, Dong Y, Poulose JT, Herasevich V, Garza JA, Gajic O (2011). Timing of the onset of acute respiratory distress syndrome: a population-based study. Respir Care.

[6] Force ADT, Ranieri VM, Rubenfeld GD, Thompson BT, Ferguson ND, Caldwell E, Fan E, Camporota L, Slutsky AS (2012). Acute respiratory distress syndrome: the Berlin definition. JAMA.

[7] Bernard GR, Artigas A, Brigham KL, Carlet J, Falke K, Hudson L, Lamy M, Legall JR, Morris A, Spragg R (1994). The American-European Consensus Conference on ARDS. Definitions, mechanisms, relevant outcomes, and clinical trial coordination. Am J Respir Crit Care Med.

[8] Kallet RH, Alonso JA, Pittet JF, Matthay MA (2004). Prognostic value of the pulmonary dead-space fraction during the first 6 days of acute respiratory distress syndrome. Respir Care.

[9] Seah AS, Grant KA, Aliyeva M, Allen GB, Bates JHT (2011). Quantifying the roles of tidal volume and PEEP in the pathogenesis of ventilator-induced lung injury. Ann Biomed Eng.

[10] Steinberg JM, Schiller HJ, Halter JM, Gatto LA, Lee HM, Pavone LA, Nieman GF (2004). Alveolar instability causes early ventilator-induced lung injury independent of neutrophils. Am J Respir Crit Care Med.

[11] Halter JM, Steinberg JM, Gatto LA, DiRocco JD, Pavone LA, Schiller HJ, Albert S, Lee HM, Carney D, Nieman GF (2007). Effect of positive end-expiratory pressure and tidal volume on lung injury induced by alveolar instability. Crit Care.

[12] Andrews PL, Shiber JR, Jaruga-Killeen E, Roy S, Sadowitz B, O’Toole RV, Gatto LA, Nieman GF, Scalea T, Habashi NM (2013). Early application of airway pressure release ventilation may reduce mortality in high-risk trauma patients: a systematic review of observational trauma ARDS literature. J Trauma Acute Care Surg.

[13] Roy S, Sadowitz B, Andrews P, Gatto LA, Marx W, Ge L, Wang G, Lin X, Dean DA, Kuhn M, Ghosh A, Satalin J, Snyder K, Vodovotz Y, Nieman G, Habashi N (2012). Early stabilizing alveolar ventilation prevents acute respiratory distress syndrome: a novel timing-based ventilatory intervention to avert lung injury. J Trauma Acute Care Surg.

[14] Roy S, Habashi N, Sadowitz B, Andrews P, Ge L, Wang G, Roy P, Ghosh A, Kuhn M, Satalin J, Gatto LA, Lin X, Dean DA, Vodovotz Y, Nieman G (2013). Early airway pressure release ventilation prevents ARDS-a novel preventive approach to lung injury. Shock.

[15] Jia X, Malhotra A, Saeed M, Mark RG, Talmor D (2008). Risk factors for ARDS in patients receiving mechanical ventilation for > 48 h. Chest.

[16] Kollisch-Singule M EB, Smith B, Ruiz C, Roy S, Meng Q, Jain S, Satalin J, Snyder K, Ghosh A, Marx W, Andrews P, Habashi N, Nieman G, Gatto LA (2014) Airway pressure release ventilation (APRV) reduces conducting airway micro-strain in lung injury. JACS 219:968–76 10.1016/j.jamcollsurg.2014.09.011 PMC4350231 25440027

[17] Pavone L, Albert S, DiRocco J, Gatto L, Nieman G (2007). Alveolar instability caused by mechanical ventilation initially damages the nondependent normal lung. Crit Care.

[18] Baumgardner JE, Markstaller K, Pfeiffer B, Doebrich M, Otto CM (2002). Effects of respiratory rate, plateau pressure, and positive end-expiratory pressure on PaO2 oscillations after saline lavage. Am J Respir Crit Care Med.

[19] Formenti F, Chen R, McPeak H, Matejovic M, Farmery AD, Hahn CE (2014). A fibre optic oxygen sensor that detects rapid PO2 changes under simulated conditions of cyclical atelectasis in vitro. Respir Physiol Neurobiol.

[20] Caltabeloti F, Monsel A, Arbelot C, Brisson H, Lu Q, Gu WJ, Zhou GJ, Auler JO, Rouby JJ (2014). Early fluid loading in acute respiratory distress syndrome with septic shock deteriorates lung aeration without impairing arterial oxygenation: a lung ultrasound observational study. Crit Care.

[21] Del Sorbo L, Slutsky AS (2011). Acute respiratory distress syndrome and multiple organ failure. Curr Opin Crit Care.

[22] ARDSnet (2000) Ventilation with lower tidal volumes as compared with traditional tidal volumes for acute lung injury and the acute respiratory distress syndrome. The Acute Respiratory Distress Syndrome Network. The New England journal of medicine 342:1301–1308 10.1056/NEJM200005043421801 10793162

[23] Tsuchida S, Engelberts D, Peltekova V, Hopkins N, Frndova H, Babyn P, McKerlie C, Post M, McLoughlin P, Kavanagh BP (2006). Atelectasis causes alveolar injury in nonatelectatic lung regions. Am J Respir Crit Care Med.

[24] Halter JM, Steinberg JM, Schiller HJ, DaSilva M, Gatto LA, Landas S, Nieman GF (2003). Positive end-expiratory pressure after a recruitment maneuver prevents both alveolar collapse and recruitment/derecruitment. Am J Respir Crit Care Med.

[25] Fan E, Wilcox ME, Brower RG, Stewart TE, Mehta S, Lapinsky SE, Meade MO, Ferguson ND (2008). Recruitment maneuvers for acute lung injury: a systematic review. Am J Respir Crit Care Med.

[26] Hodgson C, Keating JL, Holland AE, Davies AR, Smirneos L, Bradley SJ, Tuxen D (2009) Recruitment manoeuvres for adults with acute lung injury receiving mechanical ventilation. Cochrane Database of Systematic Reviews, Issue 2. Art. No.: CD006667. DOI: 10.1002/14651858.CD006667.pub2. 10.1002/14651858.CD006667.pub2 19370647

[27] Ferguson ND, Cook DJ, Guyatt GH, Mehta S, Hand L, Austin P, Zhou Q, Matte A, Walter SD, Lamontagne F, Granton JT, Arabi YM, Arroliga AC, Stewart TE, Slutsky AS, Meade MO, Investigators OT, Canadian Critical Care Trials G (2013). High-frequency oscillation in early acute respiratory distress syndrome. N Engl J Med.

[28] Young D, Lamb SE, Shah S, MacKenzie I, Tunnicliffe W, Lall R, Rowan K, Cuthbertson BH, Group OS (2013). High-frequency oscillation for acute respiratory distress syndrome. N Engl J Med.

[29] Grasso S, Stripoli T, De Michele M, Bruno F, Moschetta M, Angelelli G, Munno I, Ruggiero V, Anaclerio R, Cafarelli A, Driessen B, Fiore T (2007). ARDSnet ventilatory protocol and alveolar hyperinflation: role of positive end-expiratory pressure. Am J Respir Crit Care Med.

[30] Talmor D, Sarge T, Malhotra A, O’Donnell CR, Ritz R, Lisbon A, Novack V, Loring SH (2008). Mechanical ventilation guided by esophageal pressure in acute lung injury. N Engl J Med.

[31] Ferguson ND, Frutos-Vivar F, Esteban A, Fernandez-Segoviano P, Aramburu JA, Najera L, Stewart TE (2005). Acute respiratory distress syndrome: underrecognition by clinicians and diagnostic accuracy of three clinical definitions. Crit Care Med.

[32] Bikker IG, Leonhardt S, Reis Miranda D, Bakker J, Gommers D (2010). Bedside measurement of changes in lung impedance to monitor alveolar ventilation in dependent and non-dependent parts by electrical impedance tomography during a positive end-expiratory pressure trial in mechanically ventilated intensive care unit patients. Crit Care.

[33] Muders T, Luepschen H, Zinserling J, Greschus S, Fimmers R, Guenther U, Buchwald M, Grigutsch D, Leonhardt S, Putensen C, Wrigge H (2012). Tidal recruitment assessed by electrical impedance tomography and computed tomography in a porcine model of lung injury*. Crit Care Med.

[34] Blankman P, Hasan D, Erik G, Gommers D (2014). Detection of ‘best’ positive end-expiratory pressure derived from electrical impedance tomography parameters during a decremental positive end-expiratory pressure trial. Crit Care.

[35] Talmor D, Sarge T, O’Donnell CR, Ritz R, Malhotra A, Lisbon A, Loring SH (2006). Esophageal and transpulmonary pressures in acute respiratory failure. Crit Care Med.

[36] Loring SH, Pecchiari M, Della Valle P, Monaco A, Gentile G, D’Angelo E (2010). Maintaining end-expiratory transpulmonary pressure prevents worsening of ventilator-induced lung injury caused by chest wall constriction in surfactant-depleted rats. Crit Care Med.

[37] Gattinoni L, Chiumello D, Carlesso E, Valenza F (2004). Bench-to-bedside review: chest wall elastance in acute lung injury/acute respiratory distress syndrome patients. Crit Care.

[38] Kollisch-Singule M, Emr B, Smith B, Roy S, Jain S, Satalin J, Snyder K, Andrews P, Habashi N, Bates J, Marx W, Nieman G, Gatto LA (2014). Mechanical Breath Profile of Airway Pressure Release Ventilation: The Effect on Alveolar Recruitment and Microstrain in Acute Lung Injury. JAMA Surg.

[39] Roy SK, Emr B, Sadowitz B, Gatto LA, Ghosh A, Satalin JM, Snyder KP, Ge L, Wang G, Marx W, Dean D, Andrews P, Singh A, Scalea T, Habashi N, Nieman GF (2013). Preemptive application of airway pressure release ventilation prevents development of acute respiratory distress syndrome in a rat traumatic hemorrhagic shock model. Shock.

[40] Emr B, Gatto L, Roy S, Satalin J, Ghosh A, Snyder K, Andrews P, Habashi N, Marx W, Ge L, Wang G, Dean D, Vodovotz Y, Nieman G (2013) Airway pressure release ventilation prevents ventilator-induced lung injury in normal lungs. JAMA Surg 148:1005–12 10.1001/jamasurg.2013.3746 24026214

[41] Nieman GF, Bredenberg CE, Clark WR, West NR (1981). Alveolar function following surfactant deactivation. J Appl Physiol.

